# Swallowing capacity and gravity of the laryngotracheal aspiration risk in atypical cerebellar stroke: case report

**DOI:** 10.1590/2317-1782/20232021220en

**Published:** 2023-10-30

**Authors:** Sara Virgínia Paiva Santos, Brenda Carla Lima Araújo, Claudia Sordi, Carla Patrícia Hernandez Alves Ribeiro Cesar, Daniela da Costa Maia de Andrade, Thaisa Soares Caldas Batista, Sheila Schneiberg

**Affiliations:** 1 Programa de Pós-graduação de Ciências Aplicadas a Saúde - PPGCAS, Universidade Federal de Sergipe - UFS - Lagarto (SE), Brasil.; 2 Departamento de Fonoaudiologia, Universidade Federal de Sergipe - UFS - São Cristóvão (SE), Brasil.; 3 Departamento de Fonoaudiologia, Universidade Federal de Sergipe - UFS - Lagarto (SE), Brasil.; 4 Programa de Pós-graduação da Ciência da Saúde - PPGCS, Universidade Federal de Sergipe - UFS - São Cristóvão (SE), Brasil.; 5 Departamento de Fisioterapia, Universidade Federal de Sergipe - UFS - Lagarto (SE), Brasil.

**Keywords:** Stroke, Cerebellum, Dysphagia, Deglutition Disorders, Diagnostic Tests, Case Report

## Abstract

This case report aimed to evaluate the swallowing capacity and the severity of the risk of laryngotracheal aspiration of a 52-year-old female patient with atypical and rare stroke, with major injury in the cerebellar pathway. In order to measure swallowing capacity and risk of aspiration a routine clinical assessment used in the speech therapy clinic was performed and two valid clinical tests were used: Massey Bedside Swallowing Screen (MBSS) and Gugging Swallowing Screen (GUSS). After evaluation with the clinical tests, it was observed that the patient had reduced swallowing capacity, performance characterized as pathological, 100% dysfunction in the water swallowing test (MBSS), presence of choking, coughing, change in vocal quality and anterior escape. In the assessment of risk of aspiration with the GUSS, the patient presented moderate dysphagia and risk of laryngotracheal aspiration.This case report demonstrated that moderate dysphagia is found in a stroke patient with lesions that affect the cerebellum. Standardized and validated clinical tests such as GUSS and MBSS should also be used to assess the risk of dysphagia after stroke at ambulatory care.

## INTRODUCTION

Eating and drinking, in addition to being essential for the nutrition and conservation of a healthy, active life, are also everyone’s daily social life activities. Dysphagia is defined as a dysfunction in the swallowing process and may be present in 50% of stroke cases^(1)^, contributing to death (in more severe cases) and morbidity (in mild and moderate cases) rates^(2)^. Dysphagia may lead to nutritional deficiencies, increasing musculoskeletal deficits, performance capacity in activities of daily life, and restrictions in social participation, hampering the whole recovery process of individuals with after-effects of strokes^(3)^.

The assessment and treatment of dysphagia cases in patients who had strokes represent an important part of the clinical speech therapy routine; however, it is not common that ambulatory stroke patients are assessed for risk of dysphagia^(4)^. Despite the importance of the cerebellar network for the control and modulation of swallowing, only a few clinical studies investigate the occurrence and prevalence of dysphagia in cerebellar strokes^(5)^, especially after hospital discharge or in the ambulatory phase.

There is no agreement in the literature regarding the lesion area of strokes and dysphagia, as well as its severity and risk of aspiration. A systematic review sought to investigate the factors associated with the severity of dysphagia in patients who had strokes. The results demonstrated that despite the correlation between the score of the NIHSS (National Institute of Health Stroke Scale) and the severity of dysphagia, there was no association between the hemispheric location of the lesion from the strokes and the presence or severity of dysphagia^(6)^. Other studies reported low occurrence and prevalence of cases with dysphagia in pure strokes, the so-called CVAs (Cerebral Vascular Accidents), reaching only the brain hemispheres. Other studies in that systematic review^(6)^ attributed only the risk of dysphagia to cases of strokes that reach the brainstem and cerebellum^(6,7)^. In turn, a clinical test demonstrated that dysphagia is more severe and entails the risk of aspiration when the lesion occurs in the right hemisphere^(7)^.

Studies with electrostimulation in both animals and human beings are more conclusive on the control of swallowing and the association of dysphagia with lesions in the cerebellum and brainstem^(1,8)^. These studies demonstrate the role of the nuclei of the solitary tract in the brainstem, which, when stimulated, produce the swallowing movement, and, when injured, prevent stimuli from arriving in the upper laryngeal nerve^(8)^. A recent study with cats that had their cerebellar lobe removed demonstrated that the cerebellum modulates the activity of the muscles involved in swallowing. We observed a lower amplitude of the muscles involved in swallowing and hence in the swallowing reflex in the models with sectioned cerebellar lobes. The results of this study highlight that the lesions in the cerebellum may cause swallowing dysfunctions, leading to laryngotracheal aspirations due to the lower amplitude of all muscles involved in swallowing and late recruitment of the geniohyoid, thyroarytenoid, and parasternal muscles^(9)^. These studies in animals^(1,8,9)^ corroborate the results of studies performed in human beings using functional neuroimage and transcranial magnetic stimulation that suggest an organized network of afferent and efferent nuclei in the brainstem, capable to generate involuntary patterns of movements of chewing and swallowing, the so-called Central Pattern Generators (CPGs). In addition, these CPGs are modulated by cortical and subcortical areas, including the cerebellum^(8)^. Thus, scientific evidence leaves no doubts about the participation of the cerebellum in the control and modulation of swallowing neurophysiology.

In general, only a few studies report a routine of clinical exams of diagnostic screening for patients who had strokes, in speech therapy, in the outpatient phase, using standardized clinical tests and psychometric tested properties. Standardized and metrically validated instruments are considered more objective than non-standardized clinical assessments. Considering the lack of studies and strong neurophysiological evidence of the role of the cerebellum in swallowing, the speech therapy clinic needs to investigate the presence of dysphagia in an atypical and rare case of a cerebellar stroke in the ambulatory phase, in addition to testing the feasibility of standardized clinical tests for the diagnosis and classification of dysphagia.

Therefore, this study aimed to describe the assessment of swallowing capacity and the severity of the laryngotracheal aspiration risk in a patient with atypical strokes, with involvement of the cerebellar route.

### Clinical case presentation

This research was approved by the Research Ethics Committee (CEP) of the Center of Specialization in Speech Therapy – (CEFAC), Aracaju, Sergipe, under protocol number 013/12, CAAE: 02066812,7,0000,5538, decision number: 140,838. All participants signed a Free and Informed Consent Form – TCLE. This case report was presented following the recommendations of the international guidelines for the publication of CARE case reports^(10)^.

The participant of this study is a 52-year-old female patient, married and a teacher. The patient was seen at an outpatient clinic of speech therapy, in the capital of the state of Sergipe, referred by the outpatient physiotherapy clinic one year and five months after a stroke in the cerebellar routes. She was subjected to anamnesis to investigate the main complaint, background history, treatments, and assessment of swallowing a specialist speech therapist with experience in the area. The anamnesis reported the main complaint related to swallowing with frequent reports of chokings during sleep, cough after food ingestion, restless tongue, frequent cheek biting followed by bleeding, and constant tiredness. We observed that the patient had speech difficulty, hoarseness, left hemiparesis, atrophy of the right thigh, constant dizziness, bilateral hypoacusis, and difficulty to walk. The patient fed exclusively orally, and no restriction of consistency or restricted diet was reported; however, the patient reported feeding with much difficulty and anxiety. She described dysgeusia and impairment of eating pleasure. There was no background history of bronchopneumonia or malnutrition, but weight loss was reported after the neurological event.

The patient’s background presented a history of two ischemic strokes in an interval of five months, with reference to Systemic Arterial Hypertension and Diabetes Mellitus type I. Cerebral angiography showed severe ostial stenosis in the left vertebral artery, treated by endovascular technique. Computed tomography of the skull (CCT) without contrast was compatible with ischemic post-injury gliosis in the territory of the postero-inferior cerebellar artery (left cerebellar hemisphere). Chest X-rays exams were regular. Cerebral Angiography also showed arterial involvement, with stenosis in the left vertebral artery and ischemia in the left postero-inferior cerebellar artery.

#### Routine clinical speech therapy assessment

**Figure 1:**
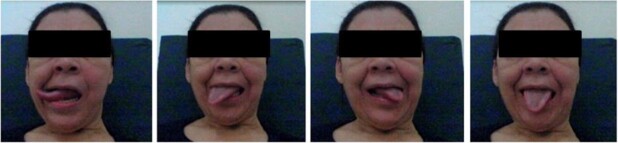
Difficulty keeping the tongue stable during exteriorization. The patient was given the following verbal command: *“Show your tongue and let it stand still without moving until I say ok”*

During the clinical speech therapy routine assessment by the physical exam, the patient was alert and oriented, presenting low touch sensitivity on the entire left hemiface, including the intra-oral region, difficulty in elevation and retraction of the palatal muscle, speech alteration characterized as dysarthria due to articulatory imprecision, monotone, hoarseness, alteration in prosody, and hypernasal resonance. We observed great damage in the oral coordination of control of the voluntary tongue movement since the patient was unable to keep her tongue still in the midline, as shown in Figure 1. In Figure 1, the patient’s tongue appears in several positions, despite the clear and explicit command for her to keep her tongue still without moving the midline.

**Figure 2:**
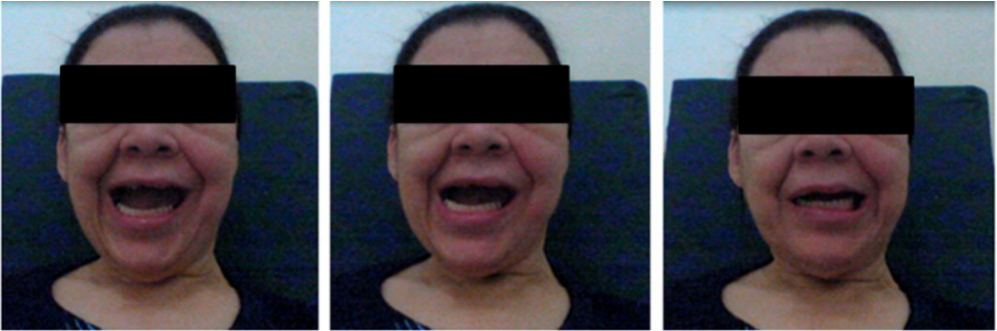
Difficulty keeping the mandible stable with the mouth open. The patient was given the following verbal command: “Make the sound /aaaaaaaa/”

The tests of oral emission of open vowels showed the involuntary lateral movement of the mandible, as shown in Figure 2. In Figure 2, the patient was given the following verbal command: “Make the sound /aaaaaaaa/”.

The oral inspection indicated a centered uvula but with damage in the elevation and retraction of the palatal muscle, worse on the left paretic side, like the GAG reflex, which occurred normally on the right side. The intraoral region on the left showed incapacity of voluntary cough start and low touch sensitivity. Such a low sensitivity is probably related to the patient’s involuntary bites, which had lesioned her internal oral cavity. Voluntary cough is an important item in speech therapy assessment in cases of suspect dysphagia since it investigates the patient’s capacity to protect and clean the airways. It was also observed the preservation of spontaneous swallowing capacity, even though it was ineffective, according to Table 1.

**Table 1:** Different findings from the routine clinic assessment (without diet)

**ASSESSMENT CLINIC OF ROUTINE SPEECH THERAPY (WITHOUT DIET)**	
**Structural assessment**	**Different findings**
Facial sensitivity	Absent in the left hemiface
Intraoral Sensitivity	Absent in the vestibule, tongue, soft palate, and oropharynx on the left side
Orofacial mobility	Hypofunction of the soft palate in retraction and elevation
Coordination	Difficulty in control in the voluntary movements of the tongue and mandible
Phonation and articulation	Dysarthria: articulatory imprecision, monopitch, hoarseness, hypernasal resonance, and difficulty in prosody
Reflexes	Adequate swallowing, Gag of difficult elicitation from the left side
Cough	Difficulty starting/performing voluntary cough

The cervical auscultation of swallowing was carried out^(11)^ before, during, and after the ingestion of each food through a stethoscope, by Littman Cardiology II, positioned on the lateral part of the junction of the larynx and the trachea, anterior to the carotid. The test with food resulted in negative cervical auscultation (regular) and the liquid intake assessment resulted in positive cervical auscultation (altered) before swallowing. The lung auscultation revealed the presence of vesicular murmurs, without alterations.

Pulse oximetry was used to monitor the risk of hypoxemia, before, during the test, and after the functional swallowing assessment. The measures were taken using a portable pulse oximeter equipment, Onyx 9500 Nonin, positioned on the patient's right index finger, after confirmation of stable signs. No significant alterations in oxygen saturation were found, and the values varied between 95 and 100%, without oscillations greater than two points during or after the tests.

The clinical routine assessment detected no cognitive-linguistic deficits such as problems of attention, understanding, or expression of oral language.

#### Standardized and validated clinical tests

Two validated clinical tests were used: the Massey Bedside Swallowing Screen (MBSS) and the Gugging Swallowing Screen (GUSS)^(11,12)^ to test the application feasibility of an outpatient service. Both tests have a universal, quantitative, and objective language for the screening of swallowing capacity and risk of aspiration.

The clinical test developed by Regina Massey and Diane Jedlika was applied to the screening of the swallowing capacity. The test is used by nurses when screening hospitalized patients with swallowing difficulty who needed specialized care by speech therapists – o MBSS – Massey Bedside Swallowing Screen^(12)^. This a fast and easy test that covers 14 items containing dichotomous answers (yes/no) addressing the following topics: level of consciousness, possible dysarthria, and/or aphasia, capacity to clench the teeth, capacity to close the lips, facial symmetry with movement, location of the tongue and uvula in the midline, gag reflex, voluntary cough, saliva swallowing (without sialorrhea), and moment of swallowing release and swallowing of liquids (one teaspoon of water and swallowing of water with 60ml). Items 13 and 14 observe the following four aspects: a) presence or absence of chokings when swallowing, b) gurgling voice, c) cough after the swallowing of water, and d) retention or not of water in the intraoral cavity. In total, twenty aspects are investigated.

Table 1 shows the first phase of the test in the routine physical exam of speech therapy and the respective results. The patient’s performance in the MBSS for the water swallowing test – items 13 (teaspoon) and 14 (60 ml) – occurred with alteration: the presence of choking, cough, alteration in vocal quality, and anterior leakage of liquid pela left lip commissure (an area corresponding to low tactile sensitivity). Thus, out of the twenty aspects investigated in the MBSS test, 16 presented some difference (80% of the test) (Table 2).

**Table 2:** Massey Bedside Swallowing Screen (MBSS)

**ASSESSMENT WITH MBSS**		
**Items**	**Yes**	**No**
**1. Level of consciousness (patient responding to commands)**	**X**	
**2. Dysarthria**	**X**	
**3. Aphasia**		**X**
**4. Able to clench teeth**		**X**
**5. Able to close lips**		**X**
**6. Facial symmetry with movements**		**X**
**7. Tongue in the midline**		**X**
**8. Uvula in the midline**	**X**	
**9. Gag reflex present**		**X**
**10. Voluntary cough**		**X**
**11. Able to swallow their saliva**	**X**	
**12. Swallowing reflex present**		**X**
**13. Teaspoon of water**		
Swallowing without choking		**X**
Gurgling voice	**X**	
Cough after water	**X**	
Water drips from the mouth	**X**	
**14. 60 ml of water**		
Swallowing without choking		**X**
Gurgling voice	**X**	
Cough after water	**X**	
Water drips from the mouth	**X**	
		

The GUSS – Gugging Swallowing Screen^(13)^ was used for the assessment of the risk of laryngotracheal aspiration, which is a fast, validated, easy and reliable test developed to detect dysphagia and risk of aspiration in patients with strokes at the acute phase. It has been used in the outpatient phase in patients with the after-effect of strokes with a suspected risk of aspiration^(14,15)^. The GUSS test is constituted of two parts: 1) preliminary assessment or indirect swallowing test, and 2) direct swallowing test, which is divided into three subparts. The four parts of the test must be applied sequentially.

The first part of the test (preliminary assessment or indirect swallowing test) may be assigned to zero (0) point if pathological or to one (1) if physiological. The patient must reach a maximum of five (5) points to advance to the second step of direct swallowing assessment. If the patient does not reach the five points in the first step, complementary assessments must be conducted, such as swallowing video fluoroscopy and recommendation of a special diet^(12)^. In the first part, the items assessed are vigilance assessment (if the alert state is preserved for fifteen minutes), the capacity of laryngeal cleansing (voluntary cough), the presence of sialorrhea, and the presence or not of vocal alteration (change in voice quality, weakness)^(13)^. The patient in this clinical case reached five points and followed the second step.

The direct swallowing test covers the assessment of swallowing for the following three different consistencies: pasty, liquid, and solid – in this order of presentation. The speech therapist must observe the following different clinical signs: swallowing inability, late swallowing, involuntary cough before, during, or after swallowing, presence of food leakage, and alteration in vocal quality. In the second step of the direct swallowing assessment, the listed values change, as follows: 0 (zero) when swallowing is not possible, 1 (one) for prolonged swallowing, and 2 (two) for regular swallowing, where the higher the total GUSS value, the better the swallowing performance. The GUSS value may vary from zero to twenty points, classifying the swallowing dysfunction (dysphagia) and assessing the risk of aspiration in four levels: severe dysfunction and high risk of aspiration (for sum values between zero and nine points) – where it is likely that the preliminary assessment or assessment with paste-like consistency could not be conducted; moderate dysfunction and risk of aspiration (from 10 to 14 points), mild dysfunction with low risk of aspiration (from 15 to 19 points), and regular/without risk of aspiration (≥ 20 points)^(13)^.

For the direct swallowing assessment with the GUSS test, the patient was tested with aligned posture (trunk/hip at 90º), cervical and trunk control, when static, and sitting with support. Table 3 shows the result of the second step of the GUSS. We observed pathological alterations in the following four assessment criteria for the liquid and solid consistencies: swallowing, involuntary cough, saliva leakage, and vocal change. The patient’s performance in the direct swallowing assessment resulted in a total sum of six (out of a total of 15 points in the second phase, according to Table 3). Based on the classification proposed in the GUSS assessment, the patient presented a total of eleven points (5 in the first step + 6 in the second step = 11), thus being classified as moderate dysphagia with a risk of aspiration.

**Table 3:** Gugging Swallowing Screen (GUSS) – Assessment of direct swallowing

**ASSESSMENT WITH GUSS**				
**Criteria**	**Food consistencies**			
	**Semisolid**	**Liquid**	**Solid**	**Total by item**
**1. Swallowing**	2	1	0	3
**2. Involuntary cough**	0	0	0	0
**3. Leakage**	1	0	0	1
**4. Voice change**	1	0	1	2
**Total:**	4	1	1	**6**

## DISCUSSION

The case report presented a patient seen at the outpatient service of speech therapy, after one year and five months after an atypical and rare stroke event with involvement of the cerebellar route. Among other observations, we detected moderate dysphagia with a risk of aspiration in a case where the anatomical lesion is specifically related to the cerebellum. This clinical diagnosis was established through routine clinical exams and validated and standardized tests.

This case study also demonstrated the feasibility of the outpatient application of two clinical screening tests, one for the MBSS swallowing capacity^(11)^ and another for the classification of dysphagia severity and screening of risk of aspiration – the GUSS^(13-15)^. Both tests detected the presence of swallowing dysfunction, confirming the suspicion raised in the assessment of the clinical speech therapy routine. Although some aspects of the clinical speech therapy routine assessment are like those of the tests applied, the assessment method used is subjective and qualitative; in addition, the dysfunction could not be classified by severity and the risk of aspiration could not be estimated accurately.

The use of objective and validated clinical tests, in addition to estimating severity and risk accurately, allows researchers and clinicians to communicate more widely among themselves and with other clinicians and researchers from other countries, sharing knowledge. However, they do not replace the clinical speech therapy assessment, which is fundamental to detecting other deficits and planning the therapeutic approach accurately. The MBSS and GUSS tests are validated and were translated into Portuguese from Portugal (without indexed publication). Currently, they have been translated into Brazilian Portuguese and are at the phase of cultural adaptation and validation by the group of authors of this case study.

Considering the complexity of an accurate diagnosis of dysphagia and the need for greater evidence for the speech therapist to treat dysphagia after hospital discharge, our research encourages further studies focusing on the application of diagnosis and classificatory dysphagia tests, such as MBSS and GUSS, at the outpatient phase, for cerebellar lesions in cases of strokes, neurodegenerative neurological dysfunctions, and progressive neurological dysfunctions of the cerebellar route. Further studies should include more significant samples for randomized and controlled clinical tests to detect the pre-and post-treatment effects of speech therapy.

It is worth emphasizing that a single clinical case is a limitation of this study for not allowing the generalization of the data obtained; however, it allows researchers and clinicians to study the topic more deeply and enlarge the knowledge of validated instruments of clinical assessment of dysphagia.

## FINAL REMARKS

Dysphagia may be present in strokes with cerebellar lesions, as demonstrated in this case report, even in patients at ambulatory phase. The standardized and validated clinical tests GUSS and MBSS can be used in the screening assessment of dysphagia in atypical cases of outpatient strokes.
