# Supplementary material for: Swallowing capacity and gravity of the laryngotracheal aspiration risk in atypical cerebellar stroke: case report
Source: Codas. 2023 Oct 30;35(5):e20210220. doi: 10.1590/2317-1782/20232021220en (PMC10688292; doi:10.1590/2317-1782/20232021220en)
Supplement: Translation

# Translation: Capacidade de deglutição e gravidade do risco de aspiração laringotraqueal no Acidente Vascular Encefálico atípico em via cerebelar: relato de caso

**DOI:** 10.1590/2317-1782/20232021220pt

**Authors:** Sara Virgínia Paiva Santos, Brenda Carla Lima Araújo, Claudia Sordi, Carla Patrícia Hernandez Alves Ribeiro Cesar, Daniela da Costa Maia de Andrade, Thaisa Soares Caldas Batista, Sheila Schneiberg

**Affiliations:** 1 Programa de Pós-graduação de Ciências Aplicadas a Saúde - PPGCAS, Universidade Federal de Sergipe - UFS - Lagarto (SE), Brasil.; 2 Departamento de Fonoaudiologia, Universidade Federal de Sergipe - UFS - São Cristóvão (SE), Brasil.; 3 Departamento de Fonoaudiologia, Universidade Federal de Sergipe - UFS - Lagarto (SE), Brasil.; 4 Programa de Pós-graduação da Ciência da Saúde - PPGCS, Universidade Federal de Sergipe - UFS - São Cristóvão (SE), Brasil.; 5 Departamento de Fisioterapia, Universidade Federal de Sergipe - UFS - Lagarto (SE), Brasil.

**Keywords:** Acidente Vascular Encefálico, Cerebelo, Transtorno de Deglutição, Teste Diagnóstico, Caso Clínico

## Abstract

Esse relato de caso teve o objetivo de avaliar a capacidade de deglutição e a gravidade do risco de aspiração laringotraqueal de uma paciente, 52 anos, com Acidente Vascular Encefálico (AVE) atípico, com comprometimento na via cerebelar. Para mensurar a capacidade de deglutição e do risco de aspiração foram utilizados a avaliação de rotina na clínica de fonoaudiologia e dois testes clínicos validados: o Massey Bedside Swallowing Screen (MBSS) e o Gugging Swallowing Screen (GUSS). Após a avaliação com os testes clínicos, foi observado que a paciente apresentou capacidade de deglutição diminuída, desempenho caracterizado como patológico e de risco, com 100% de alteração no teste de deglutição de água pelo MBSS, presença de engasgo, tosse, alteração na qualidade vocal e escape anterior de alimento. Já na avaliação do risco de aspiração com o GUSS, a paciente apresentou disfagia moderada e com risco de aspiração laringotraqueal. A disfagia pode estar presente em casos de AVE com lesão anatômica comprometendo o cerebelo e suas vias, o que sugere a importância de avaliação específica da deglutição nesses casos. Os testes GUSS e o MBSS podem ser utilizados para avaliação de casos atípicos de AVE em fase ambulatorial, com objetivos de avaliar o risco de aspiração e a capacidade de deglutição.

## INTRODUÇÃO

Comer e beber, além de serem essenciais para a nutrição e manutenção de uma vida saudável e ativa, são atividades rotineiras na vida social de qualquer pessoa. A disfagia é definida como uma disfunção no processo de deglutição e pode estar presente em 50% dos casos de Acidente Vascular Encefálico (AVE)^(1)^, contribuindo para as taxas de mortalidade (em casos mais graves) e morbidade (em casos mais leves e moderados)^(2)^. A disfagia pode levar a deficiências nutricionais, aumentando os déficits músculos-esqueléticos, capacidade e desempenho em atividades da vida diária e restrições na participação social, dificultando todo processo de reabilitação desses indivíduos com sequelas de AVE^(3)^.

A avaliação e o tratamento dos casos de disfagia em pacientes com AVE representam uma importante parte da rotina clínica da fonoaudiologia, contudo, não é comum que casos de AVE na fase ambulatorial sejam avaliados para o risco de disfagia^(4)^. Apesar da importância da rede cerebelar no controle e na modulação da deglutição, são poucos os estudos clínicos que investigaram a incidência e prevalência da disfagia no AVE cerebelar^(5)^, especialmente após alta hospitalar ou na fase ambulatorial.

Não foi encontrado consenso na literatura sobre o local da lesão do AVE e disfagia, sua gravidade e risco de aspiração. Uma revisão sistemática que buscou verificar os fatores associados à gravidade da disfagia de pacientes com AVE, demonstrou nos seus resultados que apesar de existir uma correlação entre a pontuação do NIHSS (National Institute of Health Stroke Scale) e a severidade da disfagia, não houve associação entre a localização hemisférica da lesão do AVE e a presença ou severidade da disfagia^(6)^. Outros estudos relataram baixa incidência e prevalência de casos com disfagia em AVEs puros, os chamados AVCs (Acidentes Vasculares Cerebrais), aqueles que atingem somente os hemisférios cerebrais. Outros estudos, nessa revisão sistemática^(6)^, atribuíram apenas risco de disfagia aos casos de AVE que atingem o tronco cerebral e cerebelo^(6,7)^. Já um ensaio clínico, demonstrou que a disfagia era mais grave e com risco de aspiração quando a lesão ocorria no hemisfério direito^(7)^.

Estudos com eletroestimulação em animais e em humanos são mais conclusivos sobre o controle da deglutição e na associação da disfagia com as lesões no cerebelo e tronco encefálico^(1,8)^. Esses estudos demonstram o papel dos núcleos do trato solitário no tronco cerebral, que quando estimulados produzem o movimento de deglutição, e quando lesionados impedem a chegada de estímulos ao nervo laríngeo superior^(8)^. Inclusive um recente estudo com gatos que tinham o lobo cerebelar retirado, demonstrou que o cerebelo modula a atividade dos músculos envolvidos na deglutição. Foi observado uma diminuição na amplitude dos músculos envolvidos na deglutição e consequentemente no reflexo de deglutição nos modelos com os lobos cerebelares seccionados. Os resultados desse estudo chamam a atenção que lesões no cerebelo podem causar disfunções na deglutição, levando a aspirações laringotraqueal, já que foi observado diminuição de amplitude de todos os músculos envolvidos na deglutição e atraso no recrutamento dos músculos gênio-hióideo, tireoaritenóideo e paraesternal^(9)^. Esses estudos em animais^(1,8,9)^ corroboram com os resultados de estudos realizados com humanos utilizando neuroimagem funcional e estimulação transcraniana magnética que sugerem uma rede organizada de núcleos aferentes e eferentes no tronco cerebral, capaz de gerar padrões involuntários de movimentos de mastigação e deglutição, os chamados *Central Pattern Generators* (CPGs), e que esses CPGs são modulados por áreas corticais e subcorticais, incluindo o cerebelo^(8)^. Logo, as evidências científicas não deixam dúvidas sobre a participação do cerebelo no controle e modulação da neurofisiologia da deglutição.

No geral, são poucos os estudos que relatam uma rotina de exames clínicos de triagem diagnóstica para pacientes com AVE usados na fonoaudiologia na fase ambulatorial que utilizem testes clínicos padronizados e com propriedades psicométricas testadas. Instrumentos padronizados e validados metricamente são considerados mais objetivos que avaliações clínicas não padronizadas. Devido a essa escassez de estudos e a forte evidência neurofisiológica do papel do cerebelo na deglutição, é importante para clínica fonoaudiológica investigar a presença da disfagia em um caso atípico e raro de AVE cerebelar na fase ambulatorial e testar a viabilidade de testes clínicos padronizados no diagnóstico e na classificação da disfagia.

Sendo assim, o objetivo deste estudo foi descrever a avaliação da capacidade de deglutição e da gravidade do risco de aspiração laringotraqueal de uma paciente com AVE atípico, com comprometimento na via cerebelar.

### Apresentação do caso clínico

Esta pesquisa foi aprovada pelo Comitê de Ética e Pesquisa (CEP) do Centro de Especialização em Fonoaudiologia - (CEFAC), Aracaju, Sergipe, com o número de protocolo 013/12, CAAE: 02066812.7.0000.5538, número do parecer: 140.838. A participante do estudo assinou o Termo de Consentimento Livre e Esclarecido - TCLE. A dissertação desse relato de caso seguiu as recomendações do guideline internacional para publicação de relatos de caso CARE^(10)^.

Participou desse estudo uma paciente de 52 anos, casada e professora. A paciente foi atendida numa clínica ambulatorial de fonoaudiologia, da capital do estado de Sergipe, encaminhada pela clínica ambulatorial da fisioterapia, 1 ano e 5 meses após AVE em vias cerebelares. Foi submetida à anamnese, com investigação da queixa principal, história pregressa, tratamentos e avaliação da deglutição por fonoaudiólogo especialista e com experiência na área de atuação. Na anamnese, foi relatada queixa principal relacionada à deglutição com relato de engasgos frequentes durante o sono, tosse após ingestão de alimentos, língua *“inquieta*”, frequentes mordidas na bochecha, seguidas de sangramento e cansaço constante. Foi observado que a paciente tinha dificuldade na fala, rouquidão, hemiparesia à esquerda, atrofia da coxa direita, tontura constante, hipoacusia bilateral e prejuízo na deambulação. A alimentação da paciente era realizada exclusivamente por via oral, não sendo relatada restrição de consistência ou dieta restrita, porém a paciente relatou que a alimentação era realizada com bastante dificuldade e apreensão. Foi referido disgeusia e comprometimento do prazer alimentar. Não havia história pregressa de broncopneumonia, nem de desnutrição, contudo relatou perda de peso após episódio neurológico.

Na história pregressa a paciente apresentou histórico de dois AVE isquêmicos em um intervalo de cinco meses. Referiu antecedentes de Hipertensão Arterial Sistêmica e Diabetes Mellitus tipo I. A angiografia cerebral evidenciou estenose grave ostial em artéria vertebral esquerda, tratada por técnica endovascular. A tomografia computadorizada de crânio (TCC) sem contraste mostrou-se compatível com gliose pós-injúria isquêmica em território da artéria cerebelar póstero-inferior (hemisfério cerebelar esquerdo). Exames de raio-X torácico mostraram-se normais. A Angiografia Cerebral também evidenciou comprometimento arterial, com estenose em artéria vertebral esquerda e isquemia na artéria cerebelar póstero-inferior esquerda.

#### Avaliação clínica de rotina na fonoaudiologia

Na avaliação clínica fonoaudiológica de rotina, pelo exame físico, a paciente estava alerta e orientada, apresentou uma baixa sensibilidade ao toque em toda a hemiface esquerda incluindo a região intra-oral, dificuldade na elevação e retração do palato muscular, alteração na fala caracterizada como disartria devido à imprecisão articulatória, monoaltura, rouquidão, alteração na prosódia e ressonância hipernasal. Observou-se acentuado prejuízo na coordenação oral de controle do movimento voluntário da língua, a paciente era incapaz de deixar sua língua parada na linha média, conforme mostra a Figura 1 abaixo. Notem na Figura 1, que a língua da paciente aparece em várias posições, apesar do comando ter sido claro e explícito para ela manter a língua parada sem mover na linha média.

**Figura 1:** Dificuldade de manter a língua estável na exteriorização. O comando verbal dado a paciente para o exame foi: *“Mostre a sua língua e deixe-a parada sem mover até quando eu disser ok”*

Nas provas de emissão oral de vogal aberta apresentou movimentação lateral involuntária de mandíbula, conforme mostra a Figura 2 abaixo. Na Figura 2 o comando verbal dado a paciente foi: “faça o som /aaaaaaaa/”.

**Figura 2:** Dificuldade de manter a mandíbula estável na abertura da boca. Comando verbal dado a paciente para o exame foi: “faça o som /aaaaaaaa/”

Na inspeção oral, observou-se úvula centralizada, mas com prejuízo na elevação e retração do palato muscular, pior do lado esquerdo parético, assim como para o reflexo de GAG, que, do lado direito foi desencadeado normalmente. Houve incapacidade de eliciar tosse voluntária e baixa sensibilidade ao toque na região intraoral à esquerda, essa baixa sensibilidade está relacionada provavelmente as mordidas que a paciente involuntariamente fazia, lesionando sua cavidade oral interna. A tosse voluntária é um importante item na avaliação da fonoaudiologia em casos de suspeita de disfagia, pois investiga a capacidade da paciente em proteger e limpar as vias aéreas. Também foi observada preservação da capacidade de deglutição espontânea, apesar de ineficaz, como mostra a Tabela 1.

**Tabela 1:** Achados alterados na avaliação clínica de rotina (sem dieta)

**AVALIAÇÃO CLÍNICA DE ROTINA DA FONOAUDIOLOGIA (SEM DIETA)**	
**Avaliação estrutural**	**Achados alterados**
Sensibilidade facial	Ausente na hemiface esquerda
Sensibilidade intra-oral	Ausente no vestíbulo, língua, véu palatino e orofaringe do lado esquerdo
Mobilidade orofacial	Hipofuncionamento do véu palatino na retração e elevação
Coordenação	Dificuldade de controle nos movimentos voluntários de língua e mandíbula
Fonação e articulação	Disartria: imprecisão articulatória, monopitch, rouquidão, ressonância hipernasal e dificuldade na prosódia
Reflexos	Deglutição adequada, Gag de difícil elicitação do lado esquerdo
Tosse	Dificuldade de iniciar/executar tosse voluntária

Foi realizada Ausculta cervical da deglutição^(11)^ antes, durante e após a ingestão de cada alimento, com estetoscópio de marca *Littman Cardiology II*, posicionado na parte lateral da junção da laringe e a traquéia, anterior à carótida. Obteve-se ausculta cervical negativa (normal) no teste com alimentos e positiva (alterada) na avaliação da ingesta de líquido, antes de deflagrar a deglutição. Na ausculta pulmonar foi possível observar murmúrios vesiculares presentes, sem alterações.

A oximetria de pulso foi utilizada para monitoração do risco de hipoxemia, sendo realizada antes, durante todo o teste e após a avaliação funcional da deglutição. Foi medida por equipamento específico: Oxímetro de pulso *Onyx* 9500 *Nonin*, portátil, posicionado no dedo indicador direito da paciente, após certificação de sinal estável. Não foram evidenciadas alterações significativas da saturação de oxigênio, os valores marcaram entre 95 e 100%, sem oscilações maiores que 2 pontos durante ou mesmo após os testes.

Na avaliação clínica de rotina não foram detectados déficits cognitivo-linguísticos como problemas de atenção, compreensão ou na expressão da linguagem oral.

#### Testes Clínicos Padronizados e Validados

Foram utilizados dois testes clínicos validados: o *Massey Bedside Swallowing Screen (MBSS) and the Gugging Swallowing Screen (GUSS)* ^(11,12)^. Esses testes foram utilizados com o objetivo de testar a viabilidade de sua aplicação no ambulatório e por utilizarem uma linguagem universal, quantitativa e objetiva no rastreio da capacidade de deglutição e no risco de aspiração.

Para o rastreio da capacidade de deglutição utilizou-se o teste clínico desenvolvido por Regina Massey e Diane Jedlika para enfermeiras triarem os pacientes hospitalizados com dificuldade de deglutição e que precisassem de um atendimento especializado pelos fonoaudiólogos - o MBSS - *Massey Bedside Swallowing Screen* ^(12)^. Esse teste é um teste rápido e fácil de ser aplicado, contém quatorze itens contendo respostas dicotômicas (sim/não), das quais investigam: o estado de consciência, possíveis disartria e/ou afasia, a capacidade de cerrar os dentes, a capacidade de fechar os lábios, a simetria facial com movimento, se a língua e a úvula estão na linha média, o reflexo gag, a tosse voluntária, a deglutição da saliva (sem sialorreia), o momento do disparo da deglutição e a deglutição de líquidos (uma colher de chá de água e deglutição de água com 60ml). Nos itens 13 e 14 são observados quatro aspectos: a) presença ou ausência de engasgos ao deglutir, b) voz gorgolejante, c) tosse após a deglutição de água, e d) contenção ou não da água na cavidade intraoral. Ao total são investigados vinte aspectos.

A primeira fase do teste foi testada no exame físico de rotina da fonoaudiologia e seus resultados estão na Tabela 1. O desempenho da paciente no MBSS no teste de deglutição de água, nos itens 13 (colher de chá) e 14 (60 ml), ocorreu com alteração: presença de engasgo, tosse, alteração na qualidade vocal e escape anterior de líquido pela comissura labial esquerda (local que referiu baixa sensibilidade tátil). Assim, dos vinte aspectos investigados no teste MBSS, 16 apresentaram-se alterados (80% do teste) (Tabela 2).

**Tabela 2:** Massey Bedside Swallowing Screen (MBSS)

**AVALIAÇÃO COM A MBSS**		
**Itens**	**Sim**	**Não**
**1. Estado de consciência (o paciente responde aos comandos)**	**X**	
**2. Disartria**	**X**	
**3. Afasia**		**X**
**4. Capaz de cerrar dentes**		**X**
**5. Capaz de fechar lábios**		**X**
**6. Simetria facial com movimentos**		**X**
**7. Língua na linha média**		**X**
**8. Úvula na linha média**	**X**	
**9. Reflexo gag presente**		**X**
**10. Tosse voluntária**		**X**
**11. Capaz de engolir sua própria saliva**	**X**	
**12. Reflexo de deglutição presente**		**X**
**13. Colher de chá de água**		
Engole sem engasgar		**X**
Voz gorgolejante	**X**	
Tosse depois da água	**X**	
Água escorre da boca	**X**	
**14. 60 ml de água**		
Engole sem engasgar		**X**
Voz gorgolejante	**X**	
Tosse depois da água	**X**	
Água escorre da boca	**X**	
		

Para a avaliação do risco de aspiração laringotraqueal foi utilizado o GUSS - *Gugging Swallowing Screen* ^(13)^, um teste rápido, validado, fácil e confiável desenvolvido para identificar disfagia e risco de aspiração em pacientes com AVE na fase aguda, que já vem sendo utilizado na fase ambulatorial em pacientes com sequela de AVE com suspeita de risco de aspiração^(14,15)^. O teste GUSS é composto por duas partes: 1) Avaliação preliminar ou teste indireto da deglutição, 2) Teste direto da deglutição, que é dividido em três subpartes. As quatro partes do teste devem ser aplicadas de maneira sequencial.

Na primeira parte do teste (Avaliação preliminar ou teste indireto da deglutição) pode ser atribuído o ponto zero (0) se patológico e um (1) se fisiológico. O máximo de cinco (5) pontos deve ser alcançado pelo paciente para continuar na segunda etapa de avaliação direta da deglutição. Caso o paciente não alcance os cinco pontos da primeira etapa, avaliações complementares devem ser realizadas como videofluoroscopia da deglutição e recomendação de dieta especial^(12)^. Nessa primeira parte os itens avaliados são: avaliação da vigilância (se manutenção do estado de alerta por quinze minutos), capacidade de limpeza laríngea (tosse voluntária), presença de sialorreia e presença ou não de alteração vocal (mudança na qualidade da voz, fraqueza)^(13)^. A paciente desse caso clínico alcançou cinco pontos e foi dado prosseguimento para a segunda etapa.

O teste direto de deglutição é composto pela avaliação do ato de engolir três consistências diferentes, a saber: pastosa, líquida e sólida, nessa ordem de apresentação. O fonoaudiólogo deve observar os sinais clínicos alterados: impossibilidade de deglutição, deglutição atrasada, tosse involuntária antes, durante ou após a deglutição, presença de escape do alimento e alteração na qualidade vocal. Nessa segunda etapa da avaliação da deglutição direta os valores de cotação mudam, sendo: 0 (zero) para quando a deglutição não é possível, 1 (um) para deglutição prolongada e 2 (dois) para deglutição normal, sendo que quanto maior o valor total do GUSS, melhor o desempenho da deglutição. O valor do GUSS pode variar de zero a vinte pontos, classificando a disfunção da deglutição (disfagia) e avaliando o risco de aspiração em quatro níveis: severa disfunção e alto risco de aspiração (para valores da soma entre zero e nove pontos) em que possivelmente a avaliação preliminar ou a avaliação com consistência pastosa foi impossível de ser realizada; moderada disfunção e com risco de aspiração (de 10 a 14 pontos), leve disfunção com baixo risco de aspiração (de 15 a 19 pontos) e normal/sem risco de aspiração (≥ 20 pontos)^(13)^.

Durante a avaliação da deglutição direta com o teste GUSS, a paciente foi testada com postura alinhada (tronco/quadril a 90º), tendo controle cervical e de tronco, quando estática e sentada com apoio. Na Tabela 3, está exposto o resultado da segunda etapa da GUSS. Foram observadas alterações patológicas nos quatro critérios de avaliação para as consistências líquida e sólida: deglutição, tosse involuntária, escape de saliva e mudança vocal. O somatório total correspondente ao desempenho da avaliação da deglutição direta da paciente foi seis (de um total de 15 pontos nessa segunda fase, conforme Tabela 3). De acordo com a classificação proposta na avaliação com o GUSS, a paciente apresentou um total de onze pontos (5, primeira etapa + 6 segunda etapa = 11) que a classifica como disfagia moderada com risco de aspiração.

**Tabela 3:** Gugging Swallowing Screen (GUSS) - Avaliação da deglutição direta

**AVALIAÇÃO COM A GUSS**				
**Critérios**	**Consistências dos alimentos**			
	**Semi sólido**	**Líquido**	**Sólido**	**Total por itens**
**1. Deglutição**	2	1	0	3
**2. Tosse involuntária**	0	0	0	0
**3. Escape**	1	0	0	1
**4. Mudança da voz**	1	0	1	2
**Total:**	4	1	1	**6**

## DISCUSSÃO

Nesse relato de caso foi apresentado uma paciente atendida no ambulatório de fonoaudiologia, após 1 ano e 5 meses de episódio de um AVE atípico e raro com comprometimento na via cerebelar. Entre outras observações, foi possível identificar que em um caso onde a lesão anatômica está especificamente relacionada ao cerebelo foi detectada disfagia moderada e com risco de aspiração. Esse diagnóstico clínico foi possível através de exames clínicos de rotina e de testes validados e padronizados.

Esse estudo de caso também demonstrou a viabilidade de aplicação ambulatorial de dois testes clínicos de rastreio, um para capacidade de deglutição MBSS^(11)^ e o outro para classificação da severidade da disfagia e triagem do risco de aspiração - o GUSS^(13-15)^. Ambos testes detectaram a presença da disfunção na deglutição, confirmando a suspeita durante a avaliação de rotina clínica da fonoaudiologia. Apesar da avaliação clínica de rotina da fonoaudiologia ter aspectos similares aos itens dos testes aplicados, o método de avaliação utilizado é subjetivo e qualitativo, além da disfunção não poder ser classificada pela severidade e o risco de aspiração não pode ser estimado de forma precisa.

A utilização de testes clínicos objetivos e validados, além de estimarem com precisão a severidade e risco, permitem aos pesquisadores e clínicos se comunicarem mais amplamente entre eles e com outros clínicos e pesquisadores de outros países, compartilhando conhecimento. Porém, não substituem a avaliação clínica fonoaudiológica que é fundamental para identificar outros déficits e planejar a conduta terapêutica de forma mais precisa. Os testes MBSS e GUSS são validados e foram traduzidos para o português de Portugal (sem publicação indexada). No momento, já foram traduzidos para o português do Brasil e estão na fase de adaptação cultural e validação pelo grupo de autores desse estudo de caso.

Diante da complexidade do diagnóstico preciso da disfagia e da necessidade de maior evidência da atuação do fonoaudiólogo no tratamento da disfagia após a alta hospitalar, essa pesquisa incentiva estudos futuros de aplicação dos testes diagnósticos e classificatórios da disfagia, como o MBSS e o GUSS, na fase ambulatorial, nas lesões cerebelares em casos de AVE, disfunções neurológicas neurodegenerativas e disfunções neurológicas progressivas das vias cerebelares, com amostras mais significativas e em ensaios clínicos randomizados e controlados para identificar os efeitos pré e pós-tratamento da fonoaudiologia.

Cabe ressaltar que a limitação deste estudo reside no fato de ser um relato de caso clínico, não possibilitando a generalização dos dados obtidos, porém permite ao pesquisador e ao clínico, o estudo aprofundado do tema e o conhecimento de instrumentos validados de avaliação na clínica da disfagia.

## COMENTÁRIOS FINAIS

A disfagia pode estar presente em AVE com lesões cerebelares como demonstrou este relato de caso, mesmo em um paciente na fase ambulatorial. Os testes clínicos padronizados e validados GUSS e o MBSS podem ser utilizados para avaliação de rastreio da disfagia em casos atípicos de AVE em fase ambulatorial.
